# Multifocal Nodular Fatty Sparing Mimicking Hepatic Tumors

**DOI:** 10.7759/cureus.33254

**Published:** 2023-01-02

**Authors:** Mohamed Lameir Mukhtar Hussein, Ahmad L F Yasin, Akram Twair, Adham Darweesh

**Affiliations:** 1 Radiology, Hamad Medical Corporation, Doha, QAT

**Keywords:** nodular focal fat sparing, fatty liver, hepatic pseudotumor, opposed phase mri, case report

## Abstract

Hepatic steatosis is frequent; however, it may present with unusual patterns, resulting in a diagnostic dilemma. Herein, we present a case of fatty liver and multifocal nodular hepatic lesions that were found to be due to multifocal nodular fatty sparing, which mimics metastasis or primary multifocal tumors. As the differential diagnosis of such lesions can be difficult based on ultrasound alone, the knowledge of Magnetic Resonance Imaging (MRI) findings is crucial. It enables the radiologists to make the correct diagnosis and alleviate the patient from unnecessary biopsies.

## Introduction

Hepatic steatosis is a common condition associated with obesity and types 2 diabetes, showing about 30% prevalence among adults, and is found in 70% of diabetics [1]. As this condition may be associated with serious later consequences if not treated, radiologists are asked to report it routinely. Many studies showed that after proper treatment, hepatic steatosis showed histological evidence of improvement [1].

Radiologists should be oriented regarding different focal forms or heterogeneous patterns of hepatic fatty deposition or sparing [2]. The MRI is the most accurate non-invasive modality for quantifying hepatic fat [2]. This is a case with an incidental ultrasound finding of bilobed multifocal hypoechoic liver lesions and a background of hyperechoic liver parenchyma suspicious of multifocal hepatic tumors, for which she underwent MRI, revealed multifocal Nodular Fatty Sparing.

## Case presentation

A 52-year-old female obese patient presented with abdominal pain and vomiting history with no prior illnesses, including malignancy or diabetes. Physical examination was unremarkable. Laboratory tests showed mildly elevated liver enzymes, including alanine transaminase (99 U/L) and aspartate transaminase (59 U/L). Borderline white blood cell count of about 10.3 x10^3/uL. AFP is not elevated. Ultrasound abdomen was done to assess the liver and to rule out cholecystitis.

Radiological features

**Figure 1:**
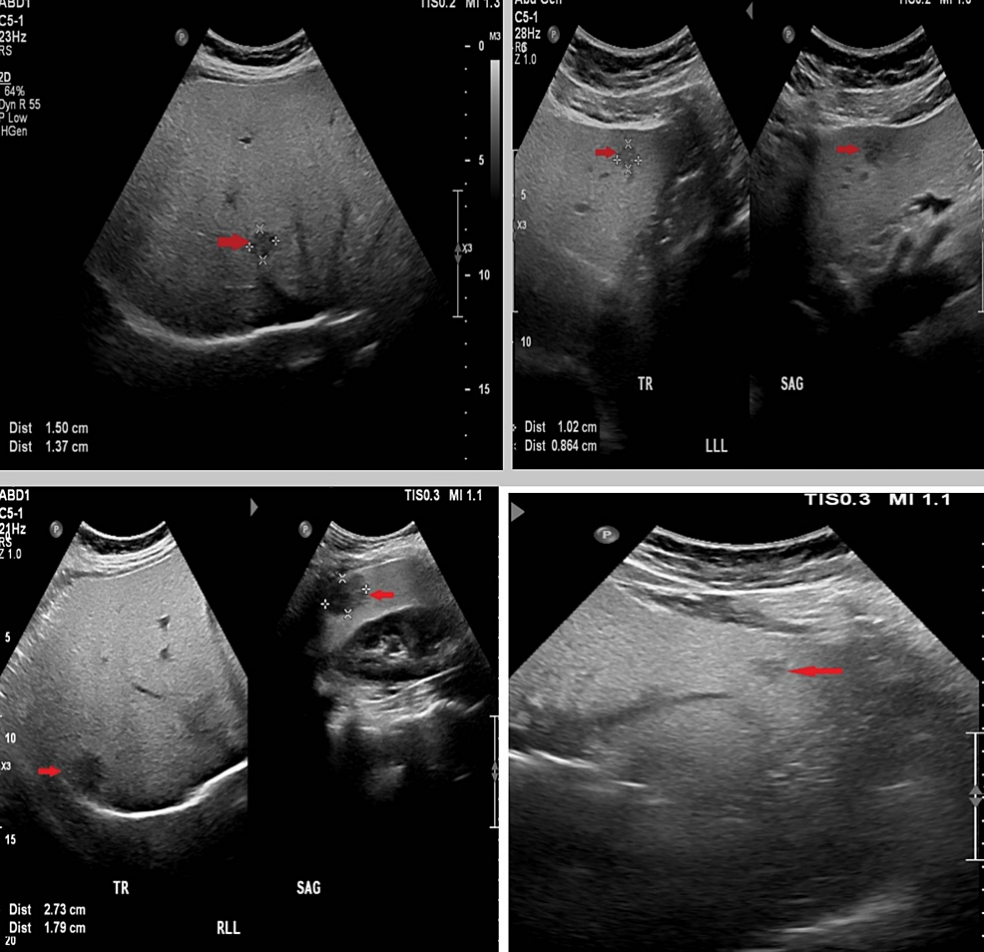
Multiple liver Ultrasound Images (A-D) reveal hyperechoic diffuse fatty liver with several focal hypoechoic masslike lesions (red arrows)

Ultrasound examination of the abdomen showed an enlarged liver measuring 20 cm in its span with increased echogenicity impressive of diffuse hepatic steatosis. In addition, there were several hypoechoic masslike focal lesions in both liver lobes; the largest lesion is in the right hepatic lobe and measures 27 x 18 mm (Figure 1). There was no intra- or extrahepatic biliary radicle dilatation. The gallbladder was normal, with no stones, wall thickening, or pericholecystic fluid. Pancreas, spleen, and kidneys were unremarkable. The ultrasound report conclusion was: Enlarged liver with multiple hypoechoic masslike focal lesions on a background of fatty liver with a differential diagnosis of hemangiomas or tumors. 

**Figure 2:**
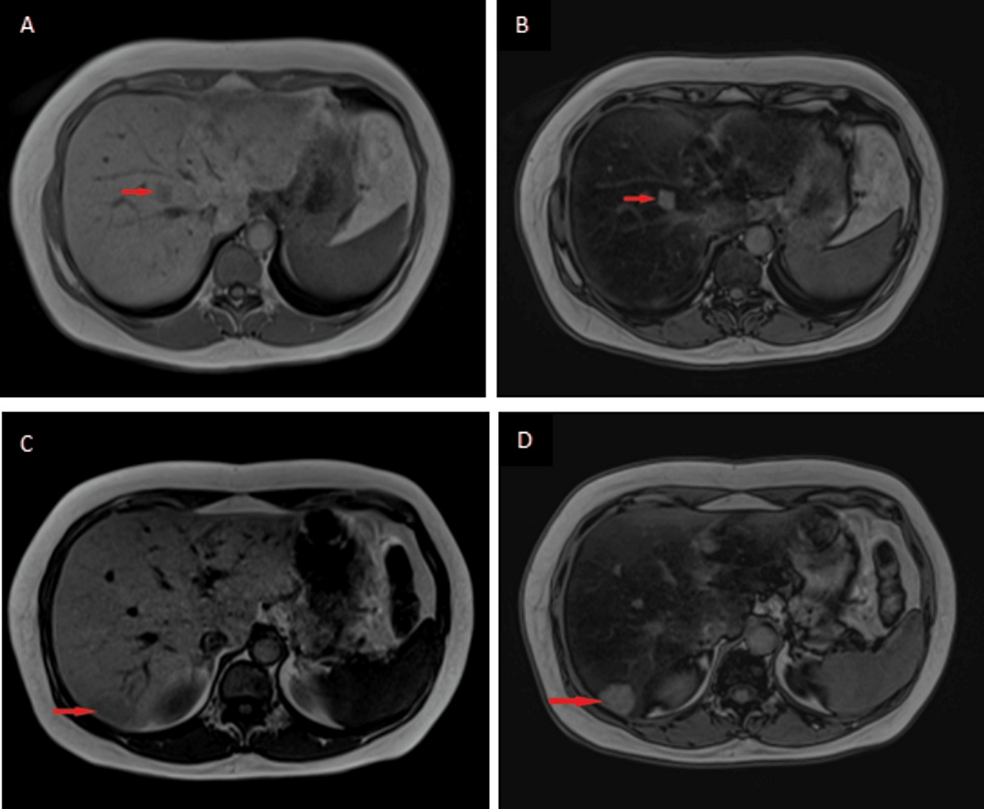
Axial T1W In-phase gradient-echo MR images (A and C) and axial Opposed-phase gradient-echo MR images (B and D) at two levels within the upper abdomen reveal a signal drop of the liver with focal areas (red arrows) of a maintained signal appearing relatively hyperintense to the fatty liver in the opposed-phase images.

**Figure 3:**
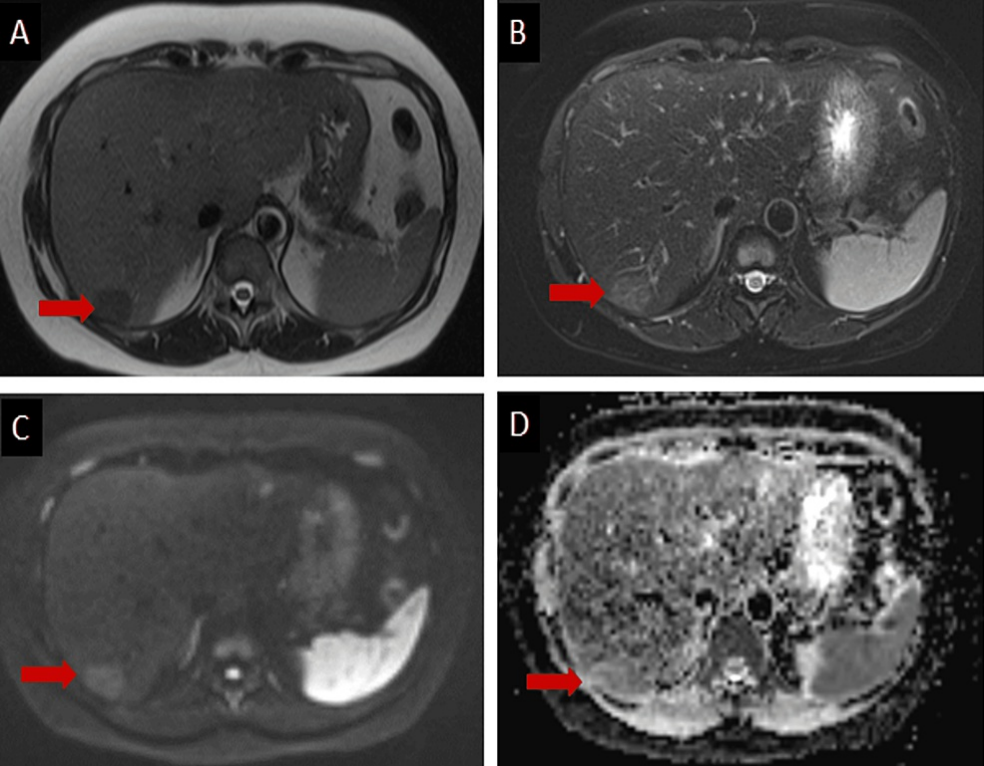
Multiple MRI sequences demonstrate the lesion in segment 6 (red arrows), which appears hypointense on T2-weighted image (A) and slightly hyperintense on T2 - weighted image fat saturation (B) with no diffusion restriction (C, diffusion-weighted images & D, ADC map).

The patient underwent an MRI liver with intravenous gadolinium to further characterize these hepatic lesions. The MRI showed hepatomegaly with diffuse fatty deposition evident by hepatic parenchymal signal drop on opposed-phase T1- weighted images (T1WI) with several focal areas of maintained normal signal appeared relatively hyperintense in opposed-phase T1WI (Figure 2), and T1WI fat saturation MRI images (Figure 3). All these focal areas are faintly visualized in the T1W image (Figure 2A) and T2W fat sat image (figure 3B) with no diffusion restriction (Figures 3C, 3D).

**Figure 4:**
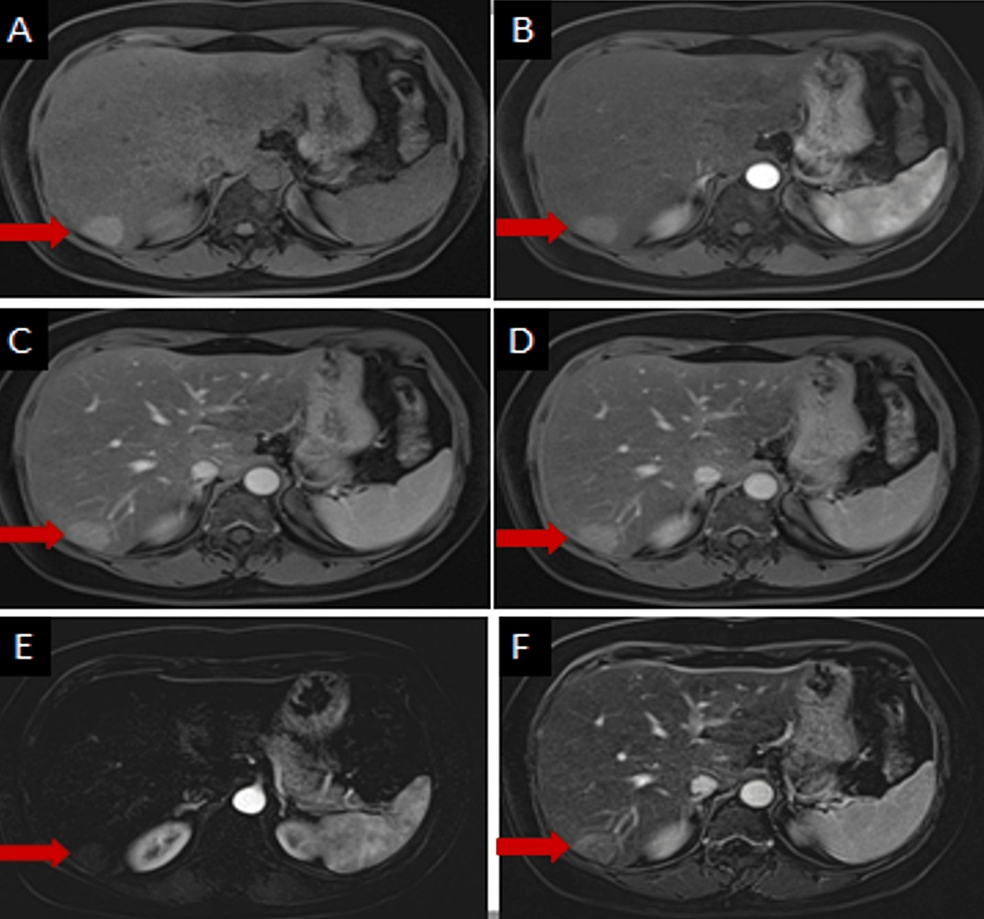
Dynamic gadolinium-enhanced T1-weighted images (A: precontrast, B: arterial, C: venous, D: delayed phases), The lesion in segment 6 appears relatively hyperintense on the precontrast fat-suppressed image (red arrow in A) with suppression of fatty signal intensity of the surrounding liver parenchyma. This lesion appears homogenously hyperintense on all three post-contrast phases with no washout relative to liver parenchyma (red arrows in B-D). Subtraction images (A: arterial & F: Venous) show mild homogenous enhancement and no washout.

On the Dynamic gadolinium-enhanced T1WI, these lesions appeared hyperintense on all three post-contrast phases and showed faint homogenous relative increased enhancement with no washout relative to liver parenchyma (Figure 4).

The patient was diagnosed with multifocal nodular fatty sparing. Her symptoms improved, and she was discharged in good condition. At 6 months follow-up, she remained asymptomatic, and a follow-up ultrasound redemonstrated of similar diffuse fatty liver with a stable number and size of the hypoechoic nodular fat-sparing areas.

## Discussion

Fatty liver is a benign entity that may show either focal or diffuse patterns. This is usually idiopathic; however, It is almost always associated with an insulin resistance syndrome, diabetes, obesity, dyslipidemia, steroid therapy, or prolonged starvation [3]. Focal fat infiltration is usually seen in segment IV adjacent to the falciform ligament or gallbladder fossa [4].

The pathophysiologic mechanism of hepatic steatosis may be related to the alteration of the vascular supply, which affects the hepatocytes adjacent to central veins and force them to accumulate lipids earlier in comparison to those in the liver periphery [5]. In contrast, some variation of the local anatomical vascularity may result in the fat-sparing areas, which occur mainly in some specific areas, including regions adjacent gallbladder and falciform ligament [1].

Focal areas of normal parenchyma in an otherwise diffuse fatty liver may mimic mass lesions. Typically, these pseudo lesions appear as hypoechoic areas against a background of hyperechoic parenchyma due to fatty infiltration. Ultrasound features that may differentiate these pseudo lesions from true abnormalities include their characteristic location in the quadrate lobe and the non-displacement of blood vessels. When these features are present, further investigation is unnecessary [4].

Hepatic fat-sparing regions may sometimes show a multinodular pattern on imaging, which mimics metastases or multifocal primary tumors [3]. MRI and mainly the “In-phase and opposed-phase” images are vital in differentiating between fatty-sparing nodules and liver tumors [6].

The biopsy is considered the gold standard diagnostic exam for focal hepatic lesions; however, this is limited by the interpretative variation and sampling error [7]. Other MRI findings that support the diagnosis of Fat-sparing areas include poor visualization on T2WI without and with fat saturation and lack of diffusion restriction on Diffusion Weighted Images (DWI). On dynamic gadolinium-enhanced T1WI, these lesions appear hyperintense on all three phases but with no washout relative to liver parenchyma, a finding which is unusual for hypervascular liver tumors [8]. Although the mechanism of this finding is unclear, we suggest that this increased relative enhancement is partly due to baseline hyperintensity on precontrast images, which resulted from paradoxical suppression of fatty signal intensity of the surrounding liver parenchyma [9]. Additionally, the fatty cells-induced narrowing or exclusion of blood vessels supplying the surrounding fatty parenchyma resulted in relative hyperenhancement of the spared areas, a finding which is rarely seen [10].

On the other hand, metastases may show high signal intensity on T2WI, an enhancing rim, surrounding fat sparing, and diffusion restriction [11]. None of these findings was found in our patient. However, we should be cautious in diagnosing focal fatty sparing of the liver in patients with a history of or high risk for cancer, as metastasis or primary hepatic tumors in patients with diffuse fatty liver can be seen as fatless liver lesions [8]. Our patient had no risk factors for liver tumors or a history of malignancy.

## Conclusions

Hepatic pseudotumors, on the background of fatty liver, appear as hypoechoic mass-like lesions on ultrasound. These are well seen on T1WI opposed phase, and T1WI fat sat images as areas with maintained normal signal may represent nodular fat sparing in appropriate clinical settings and other benign MRI features. Making an accurate diagnosis of nodular focal fat sparing prevents unnecessary biopsy or further intervention.
